# Animal Models of MS Reveal Multiple Roles of Microglia in Disease Pathogenesis

**DOI:** 10.1155/2011/383087

**Published:** 2011-11-16

**Authors:** Zhen Gao, Stella E. Tsirka

**Affiliations:** Program in Neurosciences, Department of Pharmacological Sciences, Stony Brook University, Stony Brook, NY 11794-8651, USA

## Abstract

Multiple sclerosis (MS) is a progressive inflammatory and demyelinating disease that affects more than 2.5 million people worldwide every year. Current therapies use mostly disease-modifying drugs, focusing on blocking and regulating systemic functions and the central nervous system (CNS) infiltration of immune cells; however, these therapies only attenuate or delay MS symptoms, but are not effective in halting the disease progression. More recent evidence indicated that regulation of inflammation within the CNS might be a better way to approach the treatment of the disease and microglia, the resident immune cells, may be a promising target of therapeutic studies. Microglia activation classically accompanies MS development, and regulation of microglia function changes the outcome of the disease. In this paper, we review the contributions of microglia to MS pathogenesis and discuss microglial functions in antigen presentation, cytokine release, and phagocytosis. We describe data both from animal and human studies. The significant impact of the timing, intensity, and differentiation fate of activated microglia is discussed, as they can modulate MS outcomes and potentially be critically modified for future therapeutic studies.

## 1. Background

Multiple sclerosis (MS) is a progressive autoimmune inflammatory and demyelinating disease of the central nervous system (CNS). The pathological hallmarks of MS are white matter demyelination, inflammation, axon damage, and blood-brain barrier (BBB) disruption [1–3]. The etiology of MS is still not clear, but MS is classically characterized by proinflammatory T helper (Th) cells, Th1 and Th17 infiltration into the CNS [1, 4]. However, a large number of studies suggested that MS may be initiated within the CNS in the absence of peripheral immune cell infiltration [5, 6]. Axon injury has been observed and reported very early during the development of MS, independently of lymphocyte infiltration and myelin damage [7, 8]. Oligodendrocyte (OL) apoptosis and microglia activation have been observed in MS specimens that did not yet show lymphocytic infiltration [6, 9]. Furthermore, MS is known to be attributed to viral infections, genetic background, and environment factors [2]. Therefore, MS may be not one disease, but rather a collection of different syndromes presenting themselves with inflammation and demyelination and multiple mechanisms underlying the etiology of the disease. 

Because of the complex etiology of MS, it is hard to develop a single animal model to exactly mimic the condition and symptoms of MS patients. Several animal models have been developed in different animal species to focus on different aspects of the disease. Experimental autoimmune encephalomyelitis (EAE) is the most commonly used model for MS. It is induced by immunization of mice and rats, primarily, with myelin antigens including myelin oligodendrocyte glycoprotein (MOG), myelin basic protein (MBP), and proteolipid protein (PLP) [10]. The immunization is either active (administration of the specific antigens) or passive (administration of myelin-specific T cells). Several MS features are recapitulated by EAE, including paralysis, weight loss, demyelination, and inflammation in the CNS. In the EAE model, activated myelin-specific T cells, mainly Th1 and Th17 cells, contribute to the compromise of the BBB and migrate into the CNS. In the CNS, infiltrating and local antigen presenting cells (APCs) present antigens to reactive T cells, leading to further inflammation, demyelination, and axon damage [1, 4, 10]. However, the symptoms and animal susceptibility of EAE depend on the types of immunizing antigens and strains or species of animals used. Not all myelin antigens work in every rodent strain, and different combinations may induce animal models reflecting different subtypes and clinical course of MS. H-2^U^ mice, especially the PL/J strains, are highly susceptible to MBP-induced EAE. SJL mice are susceptible to PLP-induced EAE and result in a remitting-relapsing EAE course. However, the most commonly used strain is the C57BL/6(B6) mice, as many transgenic and knockout animals are maintained on this genetic background. The C57BL/6 mice are not susceptible to MBP-induced EAE; they develop symptoms only after immunization with MOG. They can exhibit either mild/transient or severe/chronic phages depending on dosage of myelin components and immunization times [11]. For rat EAE, the most commonly used strain is the Lewis rats that are sensitive to both MBP and MOG-induced disease.

Theiler's murine encephalomyelitis virus-induced demyelinating disease (TMEV-IDD) is another inflammatory model of MS. Different from EAE, TMEV-IDD is initiated by virus infection. Responses from immune cells in this model, such as the cell types that react and the timing of reaction, may differ from EAE [12, 13]. Along similar lines, subcutaneous injection of BCG (bacillus Calmette-Guérin) has been shown to induce a delayed-type hypersensitivity (DTH) response accompanied by infiltration of macrophages and lymphocytes, the breakdown of the blood–brain barrier and immunoreactive myelin loss [14].

There are other models that induce chemical injury, such as cuprizone, lysolecithin and ethidium bromide, and result in focal demyelination in the white matter. These models are valued for studying the mechanism of demyelination/remyelination (as remyelination is initiated upon termination of the chemical injury reagent), but they cannot account for the whole picture of MS, because they are devoid of a massive leukocyte infiltration [13, 15, 16]. Compared to these models, EAE, even though it has the drawback that the disease onset is specifically due to a defined antigen presentation as opposed to MS, appears to relatively better reflect and recapitulate critical features and progressions of MS; therefore, most of the current mechanism studies and treatments for MS still mainly rely on EAE models [17]. 

Current therapies focus on blocking/regulating functions and CNS infiltration of peripheral immune cells. Application of interferon-*β* (IFN-*β*), glucocorticoids (GCs), and glatiramer acetate have been shown to be effective in slowing or delaying disease progression. However, no therapy has been shown to stop long-term progression, or to cure the disease [18, 19]. One of the drawbacks is that these therapies induce a systemic inhibition of inflammation, which may block beneficial impacts of inflammation and impair recovery. Moreover, with nonspecific inhibition, the risk of developing other adverse events, such as cancer, is increased [20, 21]. Most importantly, it is still not clear what the real initiator of the disease is, inflammation in the periphery, or damage in the CNS. Inhibiting or regulating inflammation may superficially alleviate part of symptoms, yet does not really solve the problem. 

Therefore, it may be wiser to target the local immunomodulation through enhancing the beneficial aspects of inflammation within the CNS, and at the same time promote recovery by triggering and stimulating endogenous neural cells including halting death of OLs and neurons and inducing oligogenesis by neural stem cells (NSCs). However, for this goal to be accomplished, a comprehensive understanding of the interaction between CNS and periphery immune system is required, as well as understanding the interaction between neural cells and immune cells, which still constitutes the main obstacle in MS therapeutic studies due to the complexity of the neuronal network. Nevertheless, increasing evidence supports that the resident immune cells, microglia, functioning as a bridge between the CNS and immune systems, are critical for MS pathogenesis. Regulation of microglial activation may become a new promising target for therapy [22–25]. In this review, we discuss functions of microglia in MS, EAE and other experimental studies and review evidence implicating the critical roles of microglia in the complicated neural-immune networks.

## 2. Microglia in MS

Microglia derive from myeloid origin and acquire properties including cytokine production, phagocytosis, and antigen presentation, which allow microglia to play critical roles in both innate and adaptive immunity [23, 26]. Resting microglia have a ramified morphology and can actively survey their surroundings sending out long processes to sense events occurring in their microenvironment [23]. Upon activation, microglia alter their morphology from a ramified state to assume an amoeboid shape [27]. They change expression levels of cytokines, chemokines, and surface molecules produced to respond to injury in the CNS [23]. It has been shown that microglia activation is an early event in different disease models [9, 23, 28]. Depending on their unique functions different from other resident cells in the CNS, microglia inevitably play an essential role in MS.

### 2.1. Microglia Are Required for Pathogenesis

Microglial activation was observed in both active demyelinating lesions and inflammatory nondemyelinating areas of MS brain and it persisted for the whole course of the disease [29, 30]. Activated microglia were found to be attached to half-damaged myelin sheaths [18]. Activated microglia in the active demyelinating areas were immunopositive for intracellular MBP [31]. Marik et al. showed that microglia became activated before demyelination was visualized and measurable [32]. This finding was consistent with studies using PET imaging showing that microglia activation was evident in the normal appearing white matter (NAWM), the tissue area devoid of leukocyte infiltration, demyelination, or BBB disruption [6]. Taken together, microglia activation during MS may be involved in the development and expansion of the disease. To clarify the roles of microglia activation in the pathogenesis of MS, Heppner et al. induced EAE to transgenic CD11b-HSVTK mice, in which herpes simplex virus thymidine kinase expression was driven by the monocyte/macrophage/microglia CD11b promoter, with the intention to deplete microglia activation during the disease. When these CD11b-HSVTK animals are treated with ganciclovir (GCV), all the actively proliferating cells that express the transgene (macrophages, monocytes, and microglia) die. Microglia “paralysis” (as described by the authors), mediated by ganciclovir (GCV) application, resulted in attenuation of disease severity, inflammation, and demyelination [24]. Other studies using either the macrophage/microglia inhibitory factor MIF (the tripeptide TKP) or minocycline to inhibit microglia activation also showed ameliorated EAE symptoms [22, 33, 34]. Therefore, microglia activation is a necessary component in MS pathogenesis, and inhibition of microglia activation appears to be beneficial for disease progression.

However, these results do not necessarily mean that activated microglia only contribute adversely to the disease. Studies showed that the functions of activated microglia in MS were complex and could lead to both beneficial and detrimental outcomes depending on the form and the timing of activation [22, 29, 34, 35]. Here, microglial functions in MS will be discussed in detail examining three aspects: antigen presentation, cytokine release, and phagocytosis.

### 2.2. Antigen Presentation

During MS and EAE, T helper (Th) cells are key mediators of the disease. In MS, Th1 and Th17 are shown to be the main pathogenic T cells as they promote BBB disruption, demyelination, and neurodegeneration [36, 37]. Anti-inflammatory Th2 and regulatory T cells (Treg) normally protect against autoimmunity by inducing tolerance of self-antigens, but their functions are impaired during early disease [4].

Antigen presentation is a critical process for T cell activation and modulation of their function. Antigens presented via the major histocompatibility complex (MHC-class I and MHC-class II) on the APCs are required to initiate CD8 and CD4 T cell activation. The co-stimulatory signals between CD80, CD86, and CD40 on APCs and CD28, CTLA-4, and CD40L on T cells are essential for full T cell activation. Without such costimulatory signaling, MHC-TCR (T cell receptor) binding can lead to T cell death [1, 4, 13, 38]. Dendritic cells (DCs), the classically established APCs, take the responsibility to initiate/prime naïve T cell activation [13, 39–42]. It has been reported that microglia only acquire the ability of antigen presentation upon activation, and that activated microglia favor the reactivation of primed T cells and regulate their differentiation [36, 40, 41]. 

In the MS brain, activated microglia were shown to accumulate in all plaque regions express MHC-class II molecules and to be MBP-positive [31, 43, 44], suggesting that activated microglia in MS may also regulate T cell functions and lesion formation. Studies using culture systems and animal models further confirmed this observation and elucidated possible mechanisms. *In vitro* experiments showed that microglia gave rise to DCs after stimulation by the growth factor Granulocyte-macrophage colony-stimulating factor (GM-CSF) [41]. Coculture of microglia with IFN*γ* ^+^ Th1 cells and IFN*γ* ^+^IL-17^+^ Th1/Th17 cells increased the expression of MHC class II, CD40, CD80, and CD86 on microglia. Moreover, the presence of microglia resulted in significant increase of IL-1*β*, TNF-*α*, and IL-6 expression in Th1/Th17 cultures, which further promoted the differentiation of proinflammatory Th cells. However, microglia/Th1 cocultures did not significantly affect cytokine release, suggesting that microglia may have different regulatory effects on Th1 and Th17 cells [36]. 

*In vivo*, upregulation of MHC class I and II, CD40, CD80, and CD86 expression on activated microglia was observed [36, 38, 45, 46]. During EAE, the majority of activated microglia express higher levels of MHC molecules at all stages (initiation, peak, and recovery), and their expression correlates with disease progression and T cell infiltration [36, 47]. It is well accepted that there is an upregulation of the co-stimulatory molecules CD80, CD86, and CD40 on the microglial surface, but the timing and intensity of this increased expression depend on the type of stimulation and microglial markers used. Both CD80 and CD86 are expressed on the surface of MHCII^+^F4/80^+^ microglia/macrophages in TMEV virus infected mice [45]. Murphy et al. showed in an EAE model that increased expression of co-stimulatory molecules on CD11^+^CD45^high^ activated microglia/macrophages correlated with EAE progression [36]. However, Almonlda et al. showed that only a subset of tomato lectin^+^ microglia/macrophages expressed CD86 at all stages, whereas, Iba-1^+^ microglia expressed CD86 only at the recovery stage of EAE, while there was no CD80 expression at any time point [47]. Due to lack of co-stimulatory signaling on some cells, it is possible that activated microglia may induce apoptosis of T cells.

In addition to the classically activating co-stimulatory pathways, the inhibitory signaling of B7 homolog 1 (B7-H1)/programmed death receptor-1 (PD-1) between microglia and T cells is another key regulator of T cell functions [13, 46, 48]. In acute MS lesion areas, activated microglia were B7-H1 positive [49]. Moreover, B7-H1 expression in microglia was higher than that in astrocytes and splenocytes [50]. In animal models, B7-H1^−/−^ mice or mice receiving anti-B7-H1 treatment showed worse disease symptoms, accompanied by increased demyelination and inflammation in the CNS [46, 51]. 

Although they present antigens to T cells, activated microglia do not function as mature DCs (mDCs): a high microglia/T cell ratio was required to induce proliferation of naïve T cells [52]. Activated microglia expressed only low levels of co-stimulatory molecules compared with mDCs during EAE [38, 47]. It has also been shown that activated microglia do not express fascin, a marker for DC maturation, suggesting that microglia may function as immature DCs to induce tolerance of T cells to antigens [47, 53, 54]. Collectively, microglia present antigen to T cells and regulate T cell functions throughout the whole process of MS disease. The interaction between microglia and T cells does not just promote T cell activation and differentiation, but also regulates inhibition through presenting inhibitory signaling.

### 2.3. Cytokine and Other Mediators

Cytokine release from activated microglia has been extensively studied both *in vivo* and *in vitro* using different types of stimulation [55–57]. TMEV infection, lipopolysaccharide (LPS), and IFN-*γ* induce microglial expression of proinflammatory cytokines and mediators, such as nitric oxide (NO) and TNF-*α*, to promote inflammation and antigen presentation. These “classically activated” microglia are defined as M1 microglia. The majority of activated microglia early in MS and animal models were M1 microglia. On the other hand, IL-4 and IL-13 promote “alternatively activated” microglia (M2) differentiation. M2 microglia produce anti-inflammatory mediators including IL-4 and IL-10 to induce tissue repair [23, 28, 56]. M2 microglia (CD163+) were observed throughout the acute active lesions and the hypercellular rim of chronic active lesion in MS patients [31]. Studies from EAE and other models indicated that M1 and M2 microglia may have distinct functions in MS [29, 58].

#### 2.3.1. Microglia and Neurons

Glutamate excitotoxicity-induced neurodegeneration has been described in MS and observed in the EAE model [5, 8]. EAE animals showed abnormal synaptic transmission with increased spontanous excitatory postsynaptic potential (sEPSP) frequency and a slower decay phase. Application of TNF-*α* or M1 microglia to brain slice cultures reproduced these defects. However, M1 microglia treated with anti-TNF receptor (TNFR) abolished synaptic defects suggesting that microglia regulate synaptic transmission via TNF-*α* signaling [5]. Furthermore, medium from M1 microglia significantly decreased cortical neuronal survival, whereas M2-derived medium did not have any effect [28]. Dorsal root ganglia (DRG) neurons cultured with M1 medium extended short and highly branched neurites, but those cultured with M2 medium showed bipolar morphology with longer processes. In addition, M2 microglia, together with Chondroitinase ABC (ChABC), significantly increased axon growth on inhibitory substrates of Chondroitin sulfate proteoglycan (CSPG) compared with M1 [28].

#### 2.3.2. Microglia and Oligodendrocyte/Oligogenesis

The mechanisms by which activated microglia promote demyelination and impair remyelination possibly include both induction of OL death and attenuation of oligogenesis. It has been reported that activated microglia phagocytosed myelin debris in MS and EAE [58]. Activated microglia in MS were MBP-positive, indicative of microglial phagocytosis of oligodendrocyte components; in addition, medium from M1 microglia significantly decreased survival rate of OLs in culture [31, 59].

It is not entirely clear how microglia affect oligogenesis per se, but evidence has indicated that microglia activation impaired the function of both neural stem cells (NSCs; indirect effect on oligodendrocyte precursor cells, OPCs, since OPCs derive from NSCs), and OPCs (direct effect). Enlargement of subventricular zone (SVZ) and increased proliferation of endogenous NSCs were observed during EAE and local demyelination models [34, 60]. However, temporal studies indicated that responses of NSCs were transient at the acute stage and completely lost in the chronic stages [34]. Moreover, increased NG2^+^ OPCs clustered in the SVZ but did not migrate into the white matter [60]. Experimental evidence showed that the proinflammatory microenvironment by M1 microglia contributed to the insufficient repair. Activated microglia were observed in the NSC niche in contact with the NSCs [60]. Medium from M1 microglia decreased the number of OPCs and mature OLs differentiated from NSCs. TNF-*α* inhibition dramatically down-regulated this effect, suggesting that TNF-*α* primarily mediated the detrimental effect of M1 microglia [35, 59, 61]. Inhibition of microglia by minocycline significantly increased proliferation and differentiation of OPCs in EAE [34]. Therefore, although endogenous NSCs/OPCs do respond during the disease, their reactivity is not sufficient to overcome inhibition from M1 microglia or to promote efficient oligogenesis. 

Due to the deficits of endogenous NSC/OPCs functions in MS, transplantation of NSCs has been adopted to promote recovery. Several studies showed that NSC transplantation dramatically attenuated disease severity, promoted remyelination, and inhibited inflammation [62–64]. Like endogenous NSCs, transplanted NSCs also expressed receptors for cytokine and chemokines; therefore, their migration and functions were under regulation by the inflammatory environment [65, 66]. The presence of an anti-inflammatory environment, induced by IL-10, significantly enhanced adult NSC-induced functional recovery from EAE [65]. However, transplanted NSCs can also regulate immune cell functions in a dose-dependent manner [63, 67, 68]. *In vitro* cultured neurospheres significantly inhibited proliferation of MOG-specific lymph node cells and switched their cytokine profile from Th1 to Th2 [63]. Interestingly, there is no evidence supporting the notion that transplanted NSCs may have functions that are not present in the endogenous NSCs, suggesting that numbers or intensity of NSCs during the disease might be a critical factor determining the outcomes. Even though a proinflammatory environment still inhibits functions of transplanted NSCs, because a large number of NSC cells are transplanted, a percentage of these transplanted NSCs can still survive after “dealing with” and surviving the attacks of inflammatory cells. It would then be these remaining NSCs that migrate and function to promote recovery. If this is the case, then the hypothesis that promoting a balance or homeostasis between CNS responses and immune insults is fundamental for disease recovery.

#### 2.3.3. Microglia and T Cells

In addition to the regulation of T cell activation via antigen presentation (discussed in Section 2.2), activated microglia could direct T cell migration and differentiation through modulation of the chemokine/cytokine environment.

Chemokines released from activated microglia contribute to leukocytes migration/infiltration into the CNS. *In vitro*, TMEV and IL-17A stimulated microglia expressed CCL2, CXCL3, and CCL12, which had been shown to promote the migration/infiltration of chemokine receptor-expressing leukocytes into the brain [69–71]. In MS plaques and TMEV-IDD, increased expression of CCL2, CCL4, and CCL5 was observed in activated microglia [72–75]. Inhibition of microglial activation by the tripeptide MIF/TKP significantly decreased T cell numbers in the CNS [22].

Activated microglia express cytokines required for T cell differentiation. M1 microglia stimulated by TMEV and LPS produced high levels of TNF-*α*, IL-6, and IL-12, which promoted the differentiation and expansion of Th1 and/or Th17 cells [13, 39]. Coculture of microglia and Th1/Th17 cells increased IL-1*β* and IL-6 release, which were responsible for further expansion of Th1/Th17 cells [36]. M2 microglia expressing IL-4 and IL-10 were required for Th2 and Treg differentiation [4]. TGF-*β* released from M2 directly promoted Treg differentiation and could regulate Th17 functions indirectly through inhibition of Th1 and Th2 differentiation [4, 76]. To conclude, M1 microglia promote T cell differentiation toward Th1 and Th17 fates and synergistically induce demyelination and neurodegeneration. On the other hand, M2 microglia, together with anti-inflammatory T cells (Th2 and Treg), protect the system from damage and promote recovery.

#### 2.3.4. Timing and Intensity of Microglia Activation

Both M1 proinflammatory microglia and M2 anti-inflammatory microglia contribute to MS progression, but M1 microglia are dominant early and during the whole process. This M1 prevalence is induced by differences in timing and intensity of M1 and M2 microglial activation. M1 microglia are observed in the CNS since the onset of the EAE and are sustained through the whole process. However, a delayed M2 differentiation has been described, accompanied by persisting low numbers of M2 microglia during EAE, which resulted in an imbalance between the two microglial subtypes and high M1/M2 ratio [29, 36]. Because of this delayed and lower M2 differentiation, even though the M2 population has a protective effect, it still cannot overcome the detrimental effects of the M1 population. To promote recovery through regulating microglia functions, changing the M1/M2 ratio would be a key point. In line with this, injection of IL-10/IL-13 activated M2 macrophages/monocytes significantly inhibited microglial activation and suppressed EAE symptoms presumably through rebalancing the M1/M2 ratio [29]. Similarly, the beneficial effects of minocycline on EAE symptoms might be due to its biased inhibition of M1 microglia [34, 59, 77].

Nonetheless, balancing the M1/M2 ratio does not mean that total suppression of the M1 population is good for the disease, since M1 microglia are not always bad. Butovsky et al. showed that moderate M1 microglia activation induced by IFN-*γ* could increase neurogenesis and oligogenesis, whereas excessive activation inhibited these effects [35, 61]. It is also reported that controlled microglial activation and T cell infiltration promoted recovery from optic nerve injury [78]. Therefore, M1 microglia could be beneficial for tissue recovery if the extent of their activation is controlled within a well-defined range.

#### 2.3.5. Phagocytosis

Phagocytosis of myelin debris by activated microglia was observed in MS lesions and it was an essential response to promote regeneration [25, 58]. Phagocytic ability of microglia is mediated by interactions between myelin ligands and receptors on microglia. Myelin stimulated microglial activation through the binding of complement receptor 3 (CR3), scavenger receptor AI/II (SRA), and Fc*γ* receptor (Fc*γ*R), which then induced further activation and phagocytosis [79–81]. On the other hand, signaling pathways like myelin-CD47 binding to signal regulatory protein-*α* (SIRP*α*) can down regulate microglial phagocytosis [82]. Such inhibitory pathways were originally thought to induce self-tolerance by protecting healthy cells from attacks by phagocytes. However, the expression of CD47 was reported to be significantly decreased in MS lesions, suggesting the onset of uncontrolled phagocytosis by microglia [19]. Therefore, although phagocytosis of myelin debris is beneficial for recovery, its impact on disease progression is still under debate. 

The inflammatory environment is a key regulator for microglia phagocytic function. Application of proinflammatory cytokines reduced phagocytosis by macrophages/microglia [83]. Activated microglia dramatically increased expression of the microglial triggering receptor expressed on myeloid cells 2 (TREM2) during EAE [84, 85]. Studies showed that TREM2 stimulated phagocytosis *in vitro* and promoted an anti-inflammatory state in EAE [84, 86]. Inhibition of TREM2 resulted in exacerbated EAE symptoms [87]. Furthermore, pathways mediating microglial phagocytosis, as mentioned above, including CR3/MAC-1 and CD47/SIRP*α*, were not restricted to regulating phagocytosis, but also had broad effects on microglial activation and migration [18]. It is suggested then that phagocytic function of microglia is tightly correlated with and under control of the status of microglial activation in MS. Insufficient myelin clean-up may be due to blockade of phagocytosis by an unbalanced M1/M2 ratio.

## 3. Conclusions

Microglial activation is not just a hallmark of MS, but is required for disease pathogenesis. Activated microglia differentiate into M1 and M2 microglia and contribute to both protective and detrimental aspects of the inflammatory process through antigen presentation, cytokine release, and phagocytosis (Table 1). Regulating microglial functions could both affect the level of inflammatory insults and change local responses from neural cells. However, beneficial effects will only be brought when microglial activation is well-defined in time, intensity, and direction of differentiation, which are still unclear. Further studies are required to clarify and precisely determine these parameters to fully understand the functions of microglia in MS.

## Figures and Tables

**Table 1:** Functions and outcomes of microglial activation during EAE/MS.

Function	Mediator		Timing of expression	Expression level	Outcomes
Antigen Presentation	Activating	MHC class I, MHC class II	All stages (initiation, peak and recovery)	Significantly increased	Present antigen, initiate T cell activation; Induce apoptosis without co-stimulatory molecules
		CD80, CD86, CD40,	Depends on cell markers chosen	Increased, but lower than DCs	Fully activate T cell with MHC molecules
	Inhibitory	B1-H7	Not examined	Increased	T cell activation inhibition

Cytokine/ chemokine release	Proinflammatory (M1)	IL-1*β*, TNF-*α*, IL-6, NO, IFN-*γ*, IL-12	High at onset, sustained at all stages (initiation, peak and recovery)	Dramatically increased, dominant	Induce synaptic deficits; death of neurons and OLs; Induce Th1 and Th17 differentiation; Induce disfunction of NSCs/OPCs
	Anti-inflammatory (M2)	IL-4, IL-10, TGF-*β*	Low at onset, increased at later time points	Increased, lower than M1	Promote growth of long dendrites; Induce Th2 and Treg differentiation; Increase proliferation and differentiation of NSCs/OPCs
	Chemokines	CCL2, CXCL3, CCL12, CCL4, CCL5	Varies	Increased	Regulate migration of T cells, NSCs and OPCs

Phagocytosis	Activating	CR3, SRA, Fc*γ*R, TREM2	Not examined	Increased	Facilitate recognition of myelin, remove myelin debris
	Inhibitory	SIRP*α*	Not examined	Decreased	Downregulate phagocytosis, induce self-tolerance
